# *ATG18* and *FAB1* Are Involved in Dehydration Stress Tolerance in *Saccharomyces cerevisiae*

**DOI:** 10.1371/journal.pone.0119606

**Published:** 2015-03-24

**Authors:** Gema López-Martínez, Mar Margalef-Català, Francisco Salinas, Gianni Liti, Ricardo Cordero-Otero

**Affiliations:** 1 Department of Biochemistry and Biotechnology, University Rovira i Virgili, Tarragona, Spain; 2 Institute of Research on Cancer and Ageing of Nice, University Sophia Antipolis, Nice, France; University of Tokyo, JAPAN

## Abstract

Recently, different dehydration-based technologies have been evaluated for the purpose of cell and tissue preservation. Although some early results have been promising, they have not satisfied the requirements for large-scale applications. The long experience of using quantitative trait loci (QTLs) with the yeast *Saccharomyces cerevisiae* has proven to be a good model organism for studying the link between complex phenotypes and DNA variations. Here, we use QTL analysis as a tool for identifying the specific yeast traits involved in dehydration stress tolerance. Three hybrids obtained from stable haploids and sequenced in the Saccharomyces Genome Resequencing Project showed intermediate dehydration tolerance in most cases. The dehydration resistance trait of 96 segregants from each hybrid was quantified. A smooth, continuous distribution of the anhydrobiosis tolerance trait was found, suggesting that this trait is determined by multiple QTLs. Therefore, we carried out a QTL analysis to identify the determinants of this dehydration tolerance trait at the genomic level. Among the genes identified after reciprocal hemizygosity assays, *RSM22*, *ATG18* and *DBR1* had not been referenced in previous studies. We report new phenotypes for these genes using a previously validated test. Finally, our data illustrates the power of this approach in the investigation of the complex cell dehydration phenotype.

## Introduction

Almost all yeast-based food industries are steadily expanding their use of active dry yeast (ADY) because of its greater genetic stability at room temperature and lower transport and storage costs. Unfortunately, most laboratory-developed industrial yeast strains, as well as strains isolated from industrial environments, have the biotechnological handicap of losing viability during the drying process [1]. Therefore, such strains are excluded from the commercial catalogues of yeast manufacturers, awaiting a breakthrough that would allow their desiccation to be optimized. In a previous study, we performed a genetic screen of the *Saccharomyces cerevisiae* deletion library for mutants sensitive to dehydration stress [2]. Among the genes characterized as essential for overcoming dehydration stress, only five (*SIP18*, *STF2*, *GRE1*, *YJL144w*, and *NOP6*) were found to have protective effects against dehydration stress when overexpressed [3, 4]. Recent studies investigating whether the response to desiccation involves regulation at the transcriptional and/or translational level detected changes in genes involved in lipid binding and synthesis, protein synthesis and mobility, and metabolism [5–9]. However, correlations were rare between these transcriptomic studies and genetic screens using the *S*. *cerevisiae* deletion library of mutants sensitive to dehydration stress [3, 10, 11]. In contrast, haploid strains overexpressing yeast genes encoding hydrophilic proteins (Stf2, Sip18, Gre1, Yjl144w, and Nop6), which are essential for overcoming dehydration stress, are tolerant of dry conditions [3, 4].

On the other hand, Rodríguez-Porrata *et al*.^2^ showed that the knockout mutants for four nuclear apoptotic-related genes with mitochondrial functions (*Δaif1*, *Δnuc1*, *Δcpr3*, and *Δqcr7*) were hyper-tolerant of dehydration stress. Most *S*. *cerevisiae* genes involved in qualitative traits related to their basic biology have been identified using recombinant DNA techniques. However, many phenotypes important to industrially appear to be quantitative traits that are determined by quantitative trait loci (QTLs), such as growth temperature, ethanol tolerance, acetic acid production, sporulation rate, sake aromatic compounds production, and nitrogen utilization [11–17]. Considering the large amount of genetic variability in industrial yeast, a characteristic as crucial as dehydration tolerance is likely controlled by multiple QTLs that cannot be identified by conventional molecular genetic approaches.

In this paper, we performed QTL analysis on 96 segregants derived from a cross between two haploid strains derivatives of two strains of wine yeast using statistical linkage analysis between dehydration tolerance characteristics and DNA marker genotype data. We functionally characterized two QTLs encompassing six genes involved in dehydration stress tolerance that contribute to the natural phenotypic variation in the paternal strains [11].

## Materials and Methods

### Strains and plasmids

Table 1 summarizes the yeast strains and plasmids used in this study. The *RIM15*, *BST1*, *BUD27*, *BLM10*, *YFH7*, *FAB1*, *ATG18*, *CBT1*, *MRP49*, *RSM22*, and *DBR1* genes were deleted using a short-flanking homology PCR technique in which *URA3* was the selectable marker (S1B Fig.) in the *Mat α* and *Mat a* versions of the WA (*Hyg* ^*R*^), WA (*Nat* ^*R*^), WE (*Hyg* ^*R*^), and WE (*Nat* ^*R*^) strains [18]. Degenerative primers (shown in S1 Table) were used to amplify the *URA3* deletion module from the pNSU114 plasmid [19]. Transformants were obtained using the lithium acetate transformation protocol and selected by plating on synthetic glucose media lacking uracil [18]. URA^+^ transformants were selected and restreaked to obtain single colonies, for which integrations were confirmed by PCR using the primer pair URA3Fw and GENERv, a reverse primer that anneals at the downstream region of the deleted gene (S1 Table). The URA3 module was deleted from the WE, *Δatg18* strain by transforming single mutant strains with the PCR DNA fragment obtained using the ATGufw-ATGurv primer pair from the *atg18*::*URA3* locus. The transformants, which were able to grow in the presence of 5FOA and unable to grow on SC^-ura^ medium, were further evaluated by PCR. The validated WE, *Δatg18u* strain was further transformed, as mentioned previously, to obtain the WE, *Δatg18u*, *Δfab1* strain. Haploid strains with opposite mating types were crossed on yeast peptone dextrose agar (YPDA) medium supplemented with 100 μg·ml^−1^ hygromycin B and 200 μg·ml^−1^ nourseothricin sulfate. Diagnostics for isolates from individual colonies were made with the *MAT* locus by PCR using WA (*Nat* ^*R*^) and WE (*Hyg* ^*R*^) as tester strains [20]. Recombinant DNA techniques were carried out according to standard protocols [21]. The amplification reactions contained a 1x PCR buffer, 1.25 mM dNTPs, 1.0 mM MgCl_2_, 0.3 μM of each primer, 2 ng·μl^−1^ template DNA, and 3.5 U DNA Polymerase in a total volume of 100 μl. All reactions were performed using a PCR thermal cycler for 25 cycles, as follows: denaturation, 2 min at 94°C; primer annealing, 30 s at 55°C; and primer extension, 1.5 min at 68°C.

**Table 1:** Strains and plasmid used in the study.

Strain	Relevant characteristics	References
BY4742	*MATα*, *his3Δ1*, *leu2Δ0*, *lys2Δ0*, *ura3Δ0*	[22]
DBVPG6044 (WA *Hyg* ^*R*^)	*MATa*, *ho*::*HygMX*, *ura3*::*KanMX*	[23]
DBVPG6044 (WA *Nat* ^*R*^)	*MATα*, *ho*::*NatMX*, *ura3*::*KanMX*	[23]
DBVPG6765 (WE *Hyg* ^*R*^)	*MATa*, *ho*::*HygMX*, *ura3*::*KanMX*	[23]
DBVPG6765 (WE *Nat* ^*R*^)	*MATα*, *ho*::*NatMX*, *ura3*::*KanMX*	[23]
Y12 (SA *Hyg* ^*R*^)	*MATa*, *ho*::*HygMX*, *ura3*::*KanMX*	[23]
YPS128 (NA *Hyg* ^*R*^)	*MATa*, *ho*::*HygMX*, *ura3*::*KanMX*	[23]
WE/NA	WE *Nat* ^*R*^/NA *Hyg* ^*R*^	[11]
WE/WA	WE *Nat* ^*R*^/ WA *Hyg* ^*R*^	[11]
WA/WE	WA *Nat* ^*R*^/ WE *Hyg* ^*R*^	This work
WE/SA	WE *Nat* ^*R*^/ SA *Hyg* ^*R*^	[11]
96 spores WE/NA	F1 from WE *Nat* ^*R*^/NA *Hyg* ^*R*^	[11]
96 spores WE/WA	F1 from WE *Nat* ^*R*^/ WA *Hyg* ^*R*^	[11]
96 spores WE/SA	F1 from WE *Nat* ^*R*^/ SA *Hyg* ^*R*^	[11]
WA, *Δrim15*	*MATα*, *ho*::*NatMX*, *rim15*::*URA3*	This work
WA, *Δbst1*	*MATα*, *ho*::*NatMX*, *bst1*::*URA3*	This work
WA, *Δbud27*	*MATα*, *ho*::*NatMX*, *bud27*::*URA3*	This work
WA, *Δblm10*	*MATα*, *ho*::*NatMX*, *blm10*::*URA3*	This work
WA, *Δyfh7*	*MATα*, *ho*::*NatMX*, *yfh7*::*URA3*	This work
WA, *Δfab1*	*MATα*, *ho*::*NatMX*, *fab1*::*URA3*	This work
WA, *Δatg18*	*MATα*, *ho*::*NatMX*, *atg18*::*URA3*	This work
WA, *Δcbt1*	*MATα*, *ho*::*NatMX*, *cbt1*::*URA3*	This work
WA, *Δmrp49*	*MATα*, *ho*::*NatMX*, *mrp49*::*URA3*	This work
WA, *Δrsm22*	*MATα*, *ho*::*NatMX*, *rsm22*::*URA3*	This work
WA, *Δdbr1*	*MATα*, *ho*::*NatMX*, *dbr1*::*URA3*	This work
WE, *Δrim15*	*MATα*, *ho*::*NatMX*, *rim15*::*URA3*	This work
WE, *Δbst1*	*MATα*, *ho*::*NatMX*, *bst1*::*URA3*	This work
WE, *Δbud27*	*MATα*, *ho*::*NatMX*, *bud27*::*URA3*	This work
WE, *Δblm10*	*MATα*, *ho*::*NatMX*, *blm10*::*URA3*	This work
WE, *Δyfh7*	*MATα*, *ho*::*NatMX*, *yfh7*::*URA3*	This work
WE, *Δfab1*	*MATα*, *ho*::*NatMX*, *fab1*::*URA3*	This work
WE, *Δatg18*	*MATα*, *ho*::*NatMX*, *atg18*::*URA3*	This work
WE, *Δrpl2a*	*MATα*, *ho*::*NatMX*, *rpl2a*::*URA3*	This work
WE, *Δcbt1*	*MATα*, *ho*::*NatMX*, *cbt11*::*URA3*	This work
WE, *Δmrp49*	*MATα*, *ho*::*NatMX*, *mrp49*::*URA3*	This work
WE, *Δrsm22*	*MATα*, *ho*::*NatMX*, *rsm22*::*URA3*	This work
WE, *Δdbr1*	*MATα*, *ho*::*NatMX*, *dbr1*::*URA3*	This work
WE, *Δatg18u*	*MATa*, *ho*::*HygMX*, *atg18*::*ura3*	This work
WE, *Δatg18u*, Δ*fab1*	*MATa*, *ho*::*HygMX*, *atg18*::*ura3*, *fab1*::*URA3*	This work
WA/*Δrim15* ^WE^	WA *Hyg* ^*R*^/WE, *Δrim15*	This work
WA/*Δbst1* ^WE^	WA *Hyg* ^*R*^/WE, *Δbst1*	This work
WA/*Δbud27* ^WE^	WA *Hyg* ^*R*^/WE, *Δbud27*	This work
WA/*Δblm10* ^WE^	WA *Hyg* ^*R*^/WE, *Δblm10*	This work
WA/*Δyfh7* ^WE^	WA *Hyg* ^*R*^/WE, *Δyfh7*	This work
WA/*Δfab1* ^WE^	WA *Hyg* ^*R*^/WE, *Δfab1*	This work
WA/*Δatg18* ^WE^	WA *Hyg* ^*R*^/WE, *Δatg18*	This work
WA/*Δrpl2a* ^WE^	WA *Hyg* ^*R*^/WE, *Δrpl2a*	This work
WA/*Δcbt1* ^WE^	WA *Hyg* ^*R*^/WE, *Δcbt1*	This work
WA/*Δmrp49* ^WE^	WA *Hyg* ^*R*^/WE, *Δmrp49*	This work
WA/*Δrsm22* ^WE^	WA *Hyg* ^*R*^/WE, *Δrsm22*	This work
WA/*Δdbr1* ^WE^	WA *Hyg* ^*R*^/WE, *Δdbr1*	This work
WE/*Δrim15* ^WA^	WE *Hyg* ^*R*^/WA, *Δrim15*	This work
WE/*Δbst1* ^WA^	WE *Hyg* ^*R*^/WA, *Δbst1*	This work
WE/*Δblm10* ^WA^	WE *Hyg* ^*R*^/WA, *Δblm10*	This work
WE/*Δyfh7* ^WA^	WE *Hyg* ^*R*^/WA, *Δyfh7*	This work
WE/*Δfab1* ^WA^	WE *Hyg* ^*R*^/WA, *Δfab1*	This work
WE/*Δatg18* ^WA^	WE *Hyg* ^*R*^/WA, *Δatg18*	This work
WE/*Δcbt1* ^WA^	WE *Hyg* ^*R*^/WA, *Δcbt1*	This work
WE/*Δmrp49* ^WA^	WE *Hyg* ^*R*^/WA, *Δmrp49*	This work
WE/*Δrsm22* ^WA^	WE *Hyg* ^*R*^/WA, *Δrsm22*	This work
WE/*Δdbr1* ^WA^	WE *Hyg* ^*R*^/WA, *Δdbr1*	This work
WA/*Δatg18u* ^*we*^, Δ*fab1* ^*we*^	WA *Nat* ^*R*^/WE, *Δatg18u*, *Δfab1*	This work
**Plasmid**		
pNSU114	* *	[24]

### Growth conditions and desiccation-rehydration process

Yeast strains were grown in shake flasks at 150 rpm in SC medium containing 0.17% yeast nitrogen base, 2% glucose, 0.5% (NH_4_)_2_SO_4_, and 25 mg·l^−1^ uracil. The desiccation-rehydration process and yeast viability assays were performed as previously described [25].

### Linkage analysis

Linkage analysis was performed using the rQTL software, and the LOD score was calculated using a normal model [11, 26, 27]. Briefly, the significance of a QTL was determined from permutations. For each trait and cross, we permuted the phenotype values within tetrads 1,000 times and recorded the maximum LOD score each time. A QTL was considered significant if its LOD score was greater than the 0.05 tail of the 1,000 permuted LOD scores.

### RNA isolation and cDNA synthesis

The total RNA was obtained from: WE, WA, WEΔ*atg18*, WEΔ*fab1*, WAΔ*atg18*, WAΔ*fab1*, and WA/Δ*atg18u* ^*WE*^, Δ*fab1* ^*WE*^ yeast cells using a RNA Kit according to the manufacturer’s protocol. The RNA was resuspended in 100 μL RNase-free water. The DNase I RNAase free kit was used to remove the 16 genomic DNA from the RNA preparations. The RNA was quantified with a spectrophotometer at an absorbance of 260 nm and tested for purity (by the A260/280 ratio) and integrity by denaturing gel electrophoresis. The first strand of cDNA was reverse transcribed from 1 μg total RNA from each sample using a First Strand cDNA Synthesis Kit according to the manufacturer’s protocol. An identical reaction without the reverse transcription was performed to verify the absence of genomic DNA. The cDNA was subsequently amplified by PCR using yeast strain specific couple of primers forward-reverse for: *ATG18*, *FAB1*, *ALG9* and *TAF10* genes (S1 Table).

### Real-time RT-PCR

Quantitative PCR for *ATG18* and *FAB1*, was carried out using a Real Time qPCR kit according to the manufacturer's protocol and was analysed on a Real-Time PCR Detection System. The thermal cycling was composed of an initial step at 50°C for 2 min followed by a polymerase activation step at 95°C for 10 min and a cycling step with the following conditions: 40 cycles of denaturation at 95°C for 15 s, annealing at 63°C for 1 min, and extension at 72°C for 1 min. Oligonucleotides of varying lengths produce dissociation peaks at different melting temperatures. Therefore, at the end of the PCR cycles, the PCR products were analysed using a heat dissociation protocol to confirm that a single PCR product was detected by the SYBR Green dye. The fluorescence data was acquired at the 72°C step. The threshold cycle (Ct) was calculated using a software to indicate significant fluorescence signals above the noise during the early cycles of amplification. The software calculated copy numbers for the target samples from the Ct using interpolation from the standard curve. The relative levels of expression of the target genes were measured using *ALG9* and *TAF10* mRNA as an internal control and calculated according to the 2^−ΔΔ*C*^_T_ method [28].

### Microscopy

Cultures of strains harbouring the *GFP*-tagged genes were grown to the stationary phase in SC medium. The cells were washed with 1× PBS buffer (pH 7.4) and fixed in 70% ethanol for 10 min at room temperature. Fluorescence was viewed using a fluorescence microscope. A digital camera and a software were used for image acquisition.

### Statistical analysis

To determine the statistical significance of data the results were analysed by one-way ANOVA, the Shapiro-Wilk test and the Scheffé test were carried out using a statistical software package. Statistical significance was set at *p*<0.001.

## Results

### Variation in dehydration stress tolerance in recombinant yeast populations

**Fig 1:**
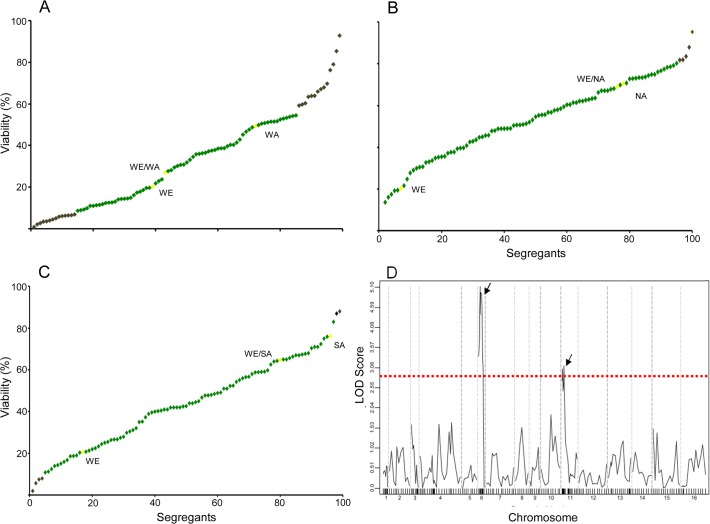
Viability rate variation after dehydration stress. Viability rate values are shown on the *y*-axis for the 96 ranked segregants of the WE x WA cross (A), WE x NA cross (B), and WE x SA cross (C). Dots indicate segregants with transgressive phenotypes (exceeding two parental standard deviations, black), parental and hybrid strains (yellow), and segregants within the phenotypic range of the parental strains (green). D) Linkage analysis for dehydration stress tolerance from WE/WA segregants. The chromosomes are displayed on the *x*-axis, and LOD viability values, according to each molecular marker across the 16 yeast chromosomes, are displayed on the *y*-axis. The significant LOD score threshold is indicated by a red line and was determined by a permutation test. The significant QTLs are indicated by arrows.

Using a colony-counting assay, desiccation tolerance was assessed for a set of three recombinant populations of 96 segregants generated from a cross of divergent *S*. *cerevisiae* isolates (WE [Wine European] x WA [West African], WE x NA [North American], and WE x SA [Sake]) previously described (S1A Fig.) [11]. The mean CFU (colony-forming units) per ml value for survival after rehydration was calculated, taking into account the viability before drying (Fig. 1A-C). The *W* value obtained from the Shapiro-Wilk test carried out with the three sets of segregants were lower than 0.5, therefore, for an α level of 0.05, the phenotypic distributions of segregants did not show a normal distribution, suggesting a polygenic contribution to cellular desiccation tolerance (Fig. 1A-C). The highest number of transgressive segregants (24%) was observed in the cross between the low dehydration stress-resistant strains WE (20.3%) and WA (49.4%) (Fig. 1A). However, when the highly sensitive WE strain was crossed with the resistant SA and NA strains (75.9% and 70.5%, respectively), approximately 5.5% of segregants exceeded the phenotypic range of their parents by at least 2 SD, criteria previously used to name these segregants as transgressive, Fig. 1B-C [29]. By running a linkage analysis using ~200 previously reported genotype markers, we evaluated whether the different genotypes correlated with the viability trend observed in the WE/WA strain segregants [11]. Only the genetic markers *Y034W*, *BST1*, *FRS2*, *RPN11*, *ROG3*, *TRP3*, and *FAS1* showed significant differences (*p*<0.005). The same analysis performed for the segregants from the WE/NA and WE/SA strains did not show any correlation between genomic region and cell viability.

### Identification of QTLs involved in dehydration tolerance

To identify the QTL intervals responsible for natural phenotypic variations in dehydration stress, linkage analysis was performed based on the cellular viability after stress induction and the genotypes of the 96 F1 segregants [11, 24]. In total, two significant regions were mapped using the marker regression model and permutation method in the WE x WA cross, allowing the identification of 15 candidate genes (Fig. 1D; Table 2). A region in chromosome XI (from 37 to 137 kb) with a peak LOD score of 3.10 was identified and after further inspections, we identified seven candidate genes (*CBT1*, *YKT6*, *FAS1*, *MRP49*, *RSM22*, *DBR1* and *AVT3*) within this QTL. In the second QTL (Chr VI, LOD 5.1), eight candidate genes (*RIM15*, *BST1*, *BUD27*, *BLM10*, *YFH7*, *FAB1*, *ATG18* and *ROG3*) were identified between 65 KB and 196 KB. After a sequence alignment, only 11 of the genes encompassed by either QTL interval (*RIM15*, *BST1*, *BUD27*, *BLM10*, *YFH7*, *FAB1*, *ATG18*, *CBT1*, *MRP49*, *RSM22* and *DBR1*) contained single-nucleotide polymorphisms (SNPs) (Table 2). Furthermore, the SNPs did not create premature stop codons in the coding sequence of the WE and WA strains. Among these genes, only *BUD27*, *FAB1*, and *CBT1* were found to be necessary for the yeast to overcome desiccation stress [3, 10, 27].

**Table 2:** The position in the genome, significance value, genes in the respective regions and the differences in the amino acid sequence for each gene in WE strain versus WA are described.

Chromosome	QTL's	Position (cM)	LOD	Gene / Position	Position of amino acid change *WA* allele→*WE* allele
VI	Y034w	65	3.85	*RIM15* /69.11	161 E → K; 240 S→G; 249 E→D; 251 T→S; 366 T→S; 399 V→A; 771 R→P; 1020 T→I; 1022 C→Y
	BST1	84	5.11	*BST1* /84.14	202 A→T; 221 N→D; 253 A→P; 432 N→D; 438 K→M; 506 Q→L; 610K→R; 636S→W; 849 D→V
				*BUD27* /90.9	32 Δ→E; 33D→Y; 75 S→F; 177 E→G; 182 D→E
	HTX10	111	4.95	*BLM10* /123.47	99 Q→R; 220 T→A; 258 G→A; 729 S→N; 759 I→V; 791 N→D; 902 C→Y; 1102 R→K; 1315 G→S; 1444 D→N; 1586 P→A; 1592 R→C; 1698 T→A; 1782 G→D; 1861 D→Y; 1900 I→V; 1971 M→I
	ARS605	136	4.93	-	
	RPN11	153	4.50	*YFH7* /159.29	109 V→I; 138 A→T; 149 V→A
	YFR016c	180	3.32	*FAB1* /184.50	120 S→N; 126 N→S; 333 A→S; 583 Δ→N; 1273 N→D; 1300 Y→H; 1524 G→E; 1604 R→M; 1780 P→S; 1878 I→M; 1882 S→A; 1884 Q→Δ
				*ATG18* /194.81	195 N→S
	ROG3	196	2.40	-	
XI	TRP3	37	2.72	*CBT1* /47.15	29 S→G; 109 T→A
	ARS1103	58	3.03	-	
	YKT6	75	2.46	-	
	FAS1	103	2.58	-	
	TP05	121	3.10	*MRP49* /133.72	131 G→R
	PIR1	142	2.34	*RSM22* /159.45	228 E→K; 474 D→S; 619 S→N
				*DBR1* /167.61	223 Q→R; 286 K→E; 325 N→D
	AVT3	173	1.16	-	

Allele without mismatch (-).

### Dissection of the QTLs associated with stress tolerance

**Fig 2:**
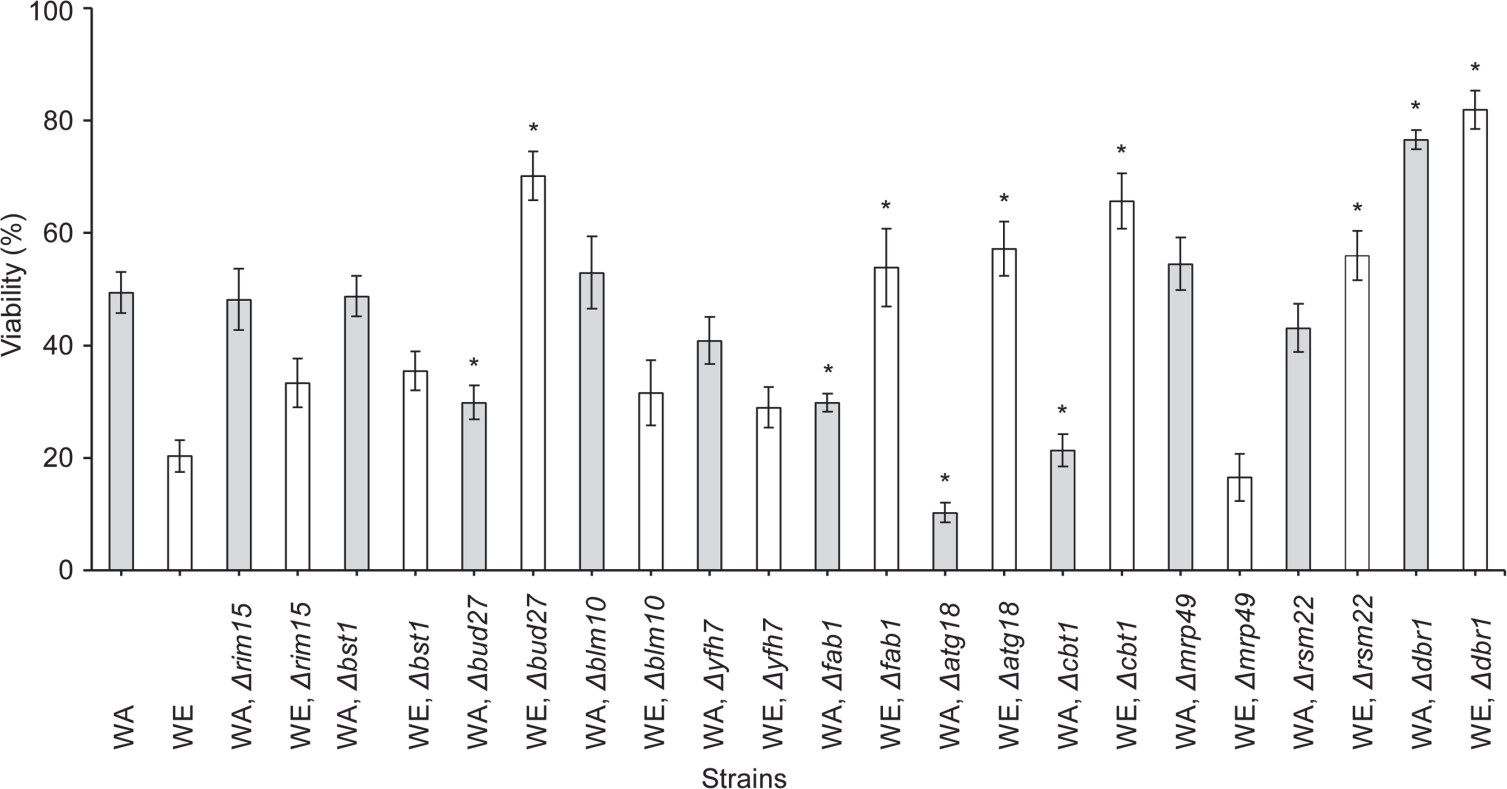
Effect of knockout haploid strains on yeast viability after DRS. The scale of viability (%) indicates the percentage of experimental values for the different strains. The values shown are means of *n* = 3 independent samples ± SD. *Significant differences (*p*≤0.01) between knockout and its own parental strains.

**Fig 3:**
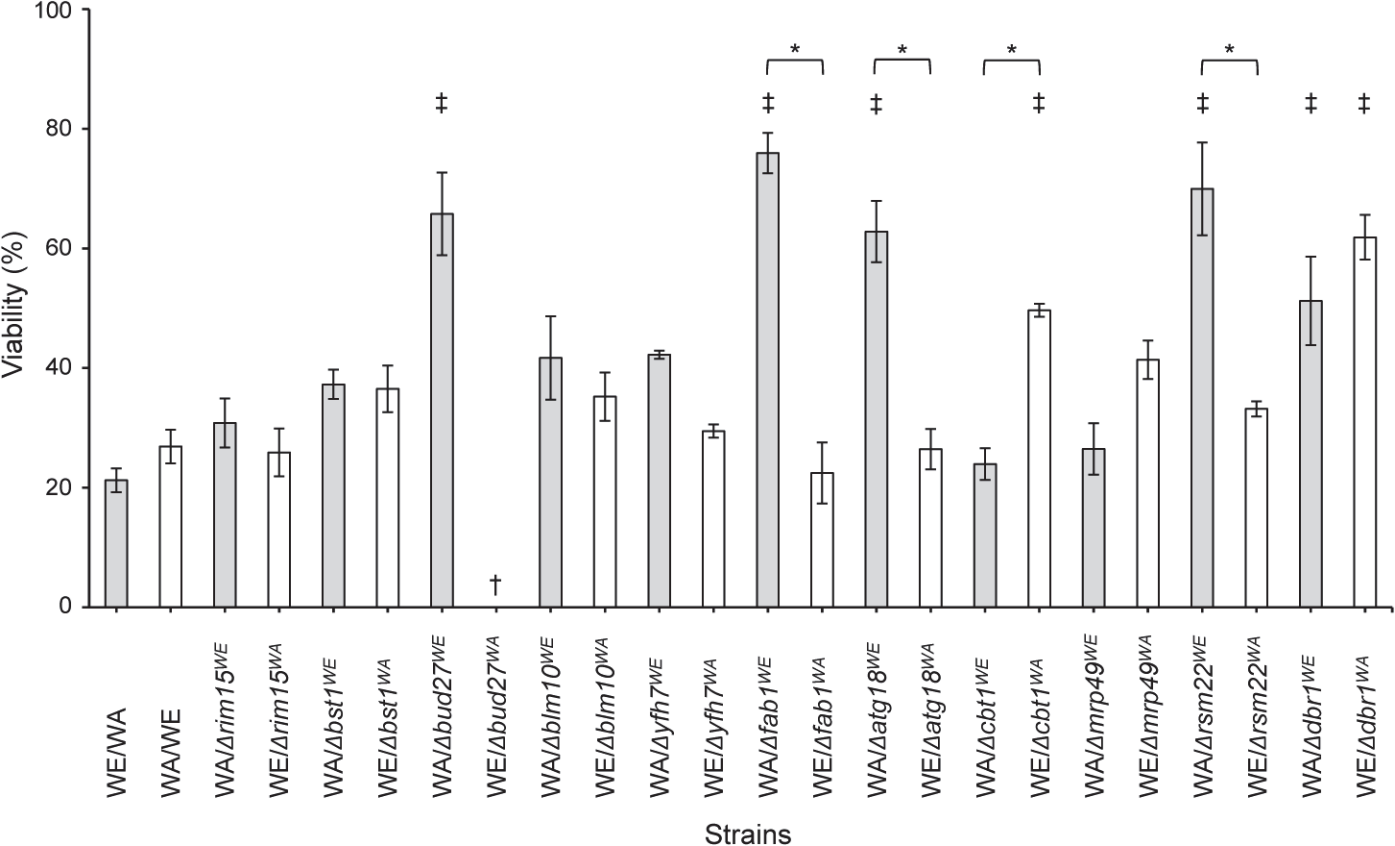
Hybrid viability after DRS. The scale of viability (%) indicates the percentage of experimental values for the different strains. The values shown are the means of at least *n* = 3 independent samples ± SD. † Non-viable strain. *Significant differences at *p*≤0.01 between hemizygous strains. ‡ Significant differences at *p*≤0.01 between the hemizygous and reference strains.

To identify causative genes within the mapped QTL intervals on chromosomes VI and XI, we generated a set of haploid strains with deletions in the candidate genes (Table 1). Then, their desiccation tolerance capacity was assessed (Fig. 2). After rehydration, four strains (WA, Δ*bud27*; WA, Δ*fab1*; WA, Δ*atg18*; and WA, Δ*cbt1*) exhibited a similar reduction in cell viability values, which were ~20% lower than in the WA strain (49%). Surprisingly, the same set of gene deletions in the WE genetic background showed the opposite effect, with viability values ~30% higher than the WT. In addition, both versions of the Δ*dbr1* strain showed significantly higher viability values after dehydration stress compared with the WT WA and WE strains (20% and 80%, respectively). Furthermore, the WE, Δ*rsm22* strain displayed 30% higher viability than its reference strain, whereas the WA, Δ*rsm22* strain had similar viability to the WA strain. The viabilities of the Δ*rim15*, Δ*bst1*, Δ*blm10*, Δ*yfh7*, and Δ*mrp49* strains were not significantly different from the WT strains, WA and WE, suggesting that these genes are not involved in desiccation-rehydration stress resistance. Therefore, two-thirds of the WE mutants enhanced dehydration stress tolerance, suggesting that the *BUD27* ^*WE*^, *FAB1* ^*WE*^, *ATG18* ^*WE*^, *CBT1* ^*WE*^, and *RSM22* ^*WE*^ alleles have a detrimental effect on the ability of the WE strain to overcome this type of stress. To confirm the impact of these alleles on dehydration stress, we used a reciprocal hemizygosity analysis (S1B Fig.) [29]. A set of isogenic hybrid strains was developed by crossing the haploid knockout strains with the complementary WA (*Nat* ^*R*^) or WE (*Hyg* ^*R*^) strain [e.g., WA (*Nat* ^*R*^) x WE Δ*rim15* (*Hyg* ^*R*^) or WA Δ*rim15* (*Hyg* ^*R*^) x WE (*Nat* ^*R*^), Table 1]. The desiccation tolerance of the hemizygous strains was measured (Fig. 3). The WA/Δ*bud27* ^*WE*^ strain showed ~40% higher viability than the WA/WE strain, which correlated with the increased viability of the WE, Δ*bud27* strain after stress induction, suggesting an adverse effect of the *BUD27* ^*WE*^ allele on stress resistance. Additionally, the WE/Δ*bud27* ^*WA*^ strain could not be obtained, suggesting a certain level of incompatibility between the *BUD27* ^*WE*^ allele and the WA genetic background. After dehydration stress induction, the hybrid strains carrying *FAB1* ^*WA*^, *ATG18* ^*WA*^, *CBT1* ^*WE*^, and *RSM22* ^*WA*^ showed viability values nearly 30% higher than the hybrids carrying *FAB1* ^*WE*^, *ATG18* ^*WE*^, *CBT1* ^*WA*^, and *RSM22* ^*WE*^ and the reference strains. The detrimental effects of the *FAB1* ^*WE*^, *ATG18* ^*WE*^, *CBT1* ^*WA*^, and *RSM22* ^*WE*^ alleles on overcoming dehydration stress were concomitant with the enhanced viability values obtained for the WE, Δ*fab1*, WE, Δ*atg18*, WA, Δ*cbt1*, and WE, Δ*rsm22* strains (Fig. 2). Furthermore, hybrids carrying either the *DBR1* ^*WE*^ or *DBR1* ^*WA*^ allele exhibited 30% higher viability than the heterozygous strains (Fig. 3). From the cell viability results for the WA, Δ*dbr1*, WE, Δ*dbr1* and heterozygous strains, a correlation can be assumed between the increasing number of *DBR1* allele copies per cell and the decreasing desiccation survival rate. The desiccation tolerances of a collection of 4,850 viable mutant haploid strains (BY4742) were previously assessed [3, 30]. For the genes above, only the Δ*rsm22* and Δ*dbr1* strains (BY4742 background) exhibited significantly higher viability values after stress induction (73% and 77%, respectively) compared with the BY4742 strain. The viability of the Δ*rim15*, Δ*bst1*, Δ*bud27*, Δ*yfh7*, Δ*fab1*, Δ*atg18*, and Δ*cbt1* strains did not significantly differ from the reference strain (34%) [2]. However, the BY4742, Δ*mrp49* strain showed 20% viability, which contrasts with the unchanging viability values for the WA, Δ*mrp49* and WE, Δ*mrp49* strains. These results confirm that *RSM22* ^*WE*^, which has 98% sequence identity to the *RSM22* ^*BY4742*^, *DBR1* ^*WA*^, *DBR1* ^*WE*^, and *DBR1* ^*BY4742*^ gene products, has a detrimental effect on dehydration stress tolerance.

### The *ATG18* ^*WE*^ allele compromises vacuole function

**Fig 4:**
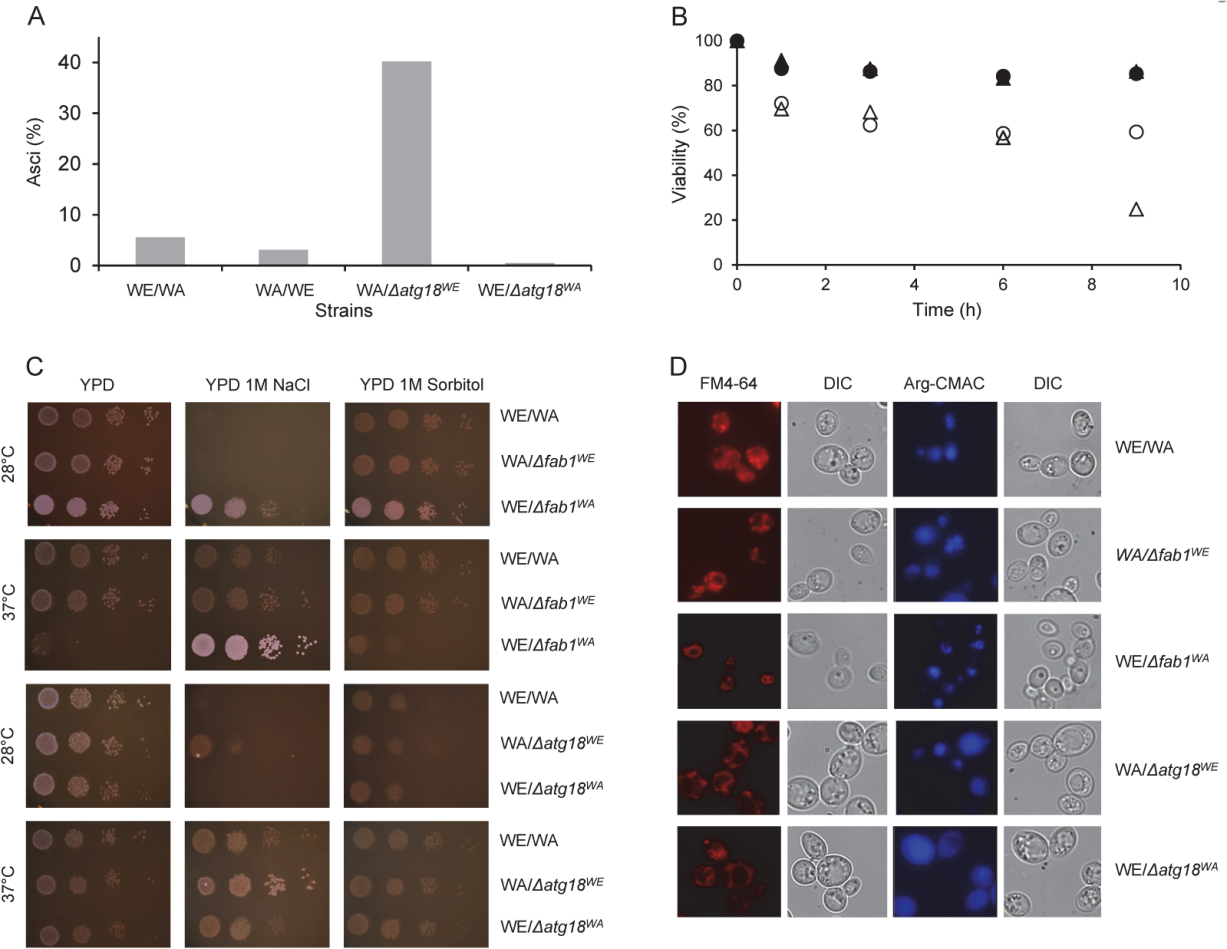
Phenotypic characterization of *ATG18* and *FAB1* alleles. A) Hemizygous diploid Δ*atg18* cells showed different sporulation patterns. After 48 hours on 1% K-acetate, the counted asci were expressed as a percentage of total cells. B) Effect of nitrogen starvation on cell viability of the Δ*atg18* strains. The hybrid WE (*Nat* ^*R*^)/WA (*Hyg* ^*R*^) (●), WA (*Nat* ^*R*^)/WE (*Hyg* ^*R*^) (▲), WE/*ATG18* ^*WA*^ (○), and WA/*ATG18* ^*WE*^ (Δ) strains were grown until the mid-log phase in SD and then moved to SD-N. Aliquots were collected and plated on YPD at the indicated times. The scale of viability (%) indicates the percentage of viable cells for the different strains against the time in starvation medium. Values are the mean of triplicate measurements, and the standard deviation was less than 15%. C) *FAB1* ^*WA*^ and *ATG18* ^*WE*^ rescue cells from ionic-hyperosmotic stress at 37°C. Serial dilutions of heterozygous and hemizygous strain cells were plated on YPD medium, YPD medium containing 1 M NaCl, and 1 M sorbitol and grown at the indicated temperatures. D) Hemizygous cells show vacuole fragmentation and vacuole acidification deficiency. Each pair of image columns show phase microscopy of the same field, which shows cells stained with FM4-64 to visualize vacuole membrane, pH vacuolar dye cell blue Arg-CMAC, and the differential interference contrast (DIC) images.

Atg18 is a key component in retrograde membrane trafficking from the vacuole to the Golgi apparatus via the endosome and is also an apparent effector and modulator of phosphatidylinositol (3,5)-bisphosphate [PtdIns(3,5)*P*_2_] [31]. It should be noted that the vacuole is responsible for amino acid storage and therefore represents the cellular reserve of nitrogen and phosphate. When yeast cells are exposed to starvation conditions, such as upon entrance into the stationary phase or during sporulation, vacuolar hydrolases are upregulated to obtain recycled nutrients through the turnover of macromolecules [32]. It follows then that malfunctions in the nutrient storage or recycling machinery are likely to compromise cell viability. Homozygous diploid Δ*atg18* is defective in autophagy prior to vacuole fusion of autophagosomes, causing the development of cell sensitivity to nitrogen starvation and non-sporulating cells [33]. The hybrid carrying A*TG18* ^*WA*^ showed 35% higher asci formation than the WE (*Nat* ^*R*^)/WA (*Hyg* ^*R*^) and WA (*Nat* ^*R*^)/WE (*Hyg* ^*R*^) strains, at 7% and 3%, respectively. However, the hybrid carrying *ATG18* ^*WE*^ showed the lowest asci formation, at 0.5% of the total cells (Fig. 4A). The wild-type and hemizygous strains were first grown to the mid-log phase and then shifted to nitrogen starvation conditions, and their viability was determined over time (Fig. 4B). The hybrid strains survived nine days of nitrogen starvation with no significant decrease in viability. In contrast, the number of viable cells for the hybrid carrying *ATG18* ^*WE*^ and the hybrid carrying *ATG18* ^*WA*^ decreased by up to 60% and 20%, respectively, over the same time period. Additionally, Δ*atg18* cells exhibited phenotypic defects, including non-acidic and conspicuous vacuoles and the loss of osmotic stress tolerance [34]. To determine putative changes in vacuole morphologies, samples of aerated wild-type, WA/Δ*atg18* ^*WE*^, and WE/Δ*atg18* ^*WA*^ cells in the stationary phase were analysed by fluorescence microscopy using FM4-64 and the blue fluorescent dye Arg-CMAC, which accumulates in acidic vacuoles (Fig. 4D). Both Δ*atg18* hemizygous strains had larger vacuoles than the WE/WA cells, but the hybrid carrying *ATG18* ^*WE*^ showed abnormal vacuolar acidification compared with the hybrid carrying *ATG18* ^*WA*^ and the WE/WA strains. To assess the consequences of the *ATG18* ^*WE*^ allele, the osmotic sensitivity was tested when the cells were grown on media containing 1 M NaCl or 1 M sorbitol at 28°C and 37°C (Fig. 4C). On the 1 M NaCl plates, the hybrid carrying *ATG18* ^*WA*^ showed better growth performance at 37°C and 28°C relative to the hybrid carrying *ATG18* ^*WE*^. No significant growth differences were exhibited between hybrids for the other serial dilutions grown on YPD and 1 M sorbitol at 37°C and 28°C. The data indicates that *ATG18* ^*WE*^ may not provide adequate nutrient storage to tolerate starvation conditions, thereby inducing both low cell viability under nitrogen starvation conditions and impaired asci formation. The *ATG18* ^*WE*^ allele was more sensitive to osmotic stress at high temperatures than the *ATG18* ^*WA*^ allele, which correlated with the differences in dehydration tolerance observed for these alleles. Furthermore, the ionic osmotic sensitivity showed by the hybrids carrying either the *ATG18* ^*WA*^ or the *ATG18* ^*WE*^ allele reverted to a resistant phenotype when the cells were grown at a high temperature.

### The *FAB1* ^*WE*^ allele enhances osmotic ionic stress tolerance

Retrograde membrane traffic from the vacuole to the Golgi apparatus via the endosome depends on PtdIns(3,5)*P*_2_.[35, 36]. The kinase FAB1p generates PtdIns(3,5)*P*_2_ via phosphatidylinositol (3)-phosphate phosphorylation [37, 38]. Abnormal levels of PtdIns(3,5)*P*_2_ were observed in Δ*atg18* yeast cells, suggesting that Atg18 is an inhibitor of the Fab1 kinase [39]. Yamamoto *et al*. [34] suggested that *fab1* mutations in yeast cells cause aberrant chromosome segregation, defects in cell surface integrity, and deficiencies in vacuole morphology and function. To determine the incidence of *FAB1* alleles in vacuole activity, WA/Δ*fab1* ^*WE*^ and WE/Δ*fab1* ^*WA*^ cells were grown on medium containing 1 M NaCl or 1 M sorbitol at 28°C and 37°C (Fig. 4C). The hybrid carrying *FAB1* ^*WE*^ grew on 1 M NaCl at 28°C, whereas the hybrid carrying *FAB1* ^*WA*^ and the WE/WA strain did not. However, all of the strains grew similarly on 1 M sorbitol. At 37°C, the hybrid carrying *FAB1* ^*WE*^ was osmoremediated on 1 M NaCl but was not recovered on 1 M sorbitol. The data indicates that ionic osmotic stress rescues the growth of *FAB1* ^*WE*^ hemizygous cells at this non-permissive temperature. The vacuolar morphology and activity of hybrid-carrying *FAB1* ^*WA*^ or *FAB* ^*WE*^ in the stationary phase were analysed using FM4-64 and Arg-CMAC dyes, respectively (Fig. 4D). The vacuolar acidity Arg-CMAC dye profile of the hemizygote cells was similar to that of the reference cells. However, Arg-CMAC and FM4-64 staining revealed vacuolar fragmentation in the hybrid carrying *FAB1* ^*WE*^, which contrasts with the single large vacuole per cell observed in both the hybrid carrying *FAB1* ^*WA*^ and the WE/WA strain. The *FAB1* ^*WE*^ allele is more sensitive than the *FAB1* ^*WA*^ allele to osmotic stress at high temperatures, which correlates with the differences in dehydration tolerance observed for these alleles. Alternatively, an isogenic strain was developed by crossing the haploid double knockout strain WE, Δ*atg18u*, Δ*fab1* with the complementary WA (*Nat* ^*R*^) strain (Table 1). The WA/Δ*atg18u* ^*we*^, Δ*fab1* ^*we*^ strain showed ~60% higher viability than the WA/WE strain, which was correlated with the increase in viability of the WE, Δ*atg18u*, Δ*fab1* strain after dehydration stress, which showed 65% viability (data not shown). Surprisingly, the double knockout WA, Δ*atg18u*, Δ*fab1* strain could not be obtained. To exclude putative artificial regulatory effect of the deletions over the genes *ATG18* or *FAB1*, which are in the same chromosome at a distance of 3.5 kb, we quantified their expression in samples from WA; WE; WA, Δ*fab*1; WA, Δ*atg18*; WE, Δ*fab*1; WE, Δ*atg18* and WA/Δ*atg18u* ^*we*^, Δ*fab*1 strains (S2 Fig.). Our data showed no statistically significant differences between the controls and the strain samples in the expression of any of the tested genes.

### The *CBT1* and *RSM22* alleles do not show respiratory deficiencies

**Fig 5:**
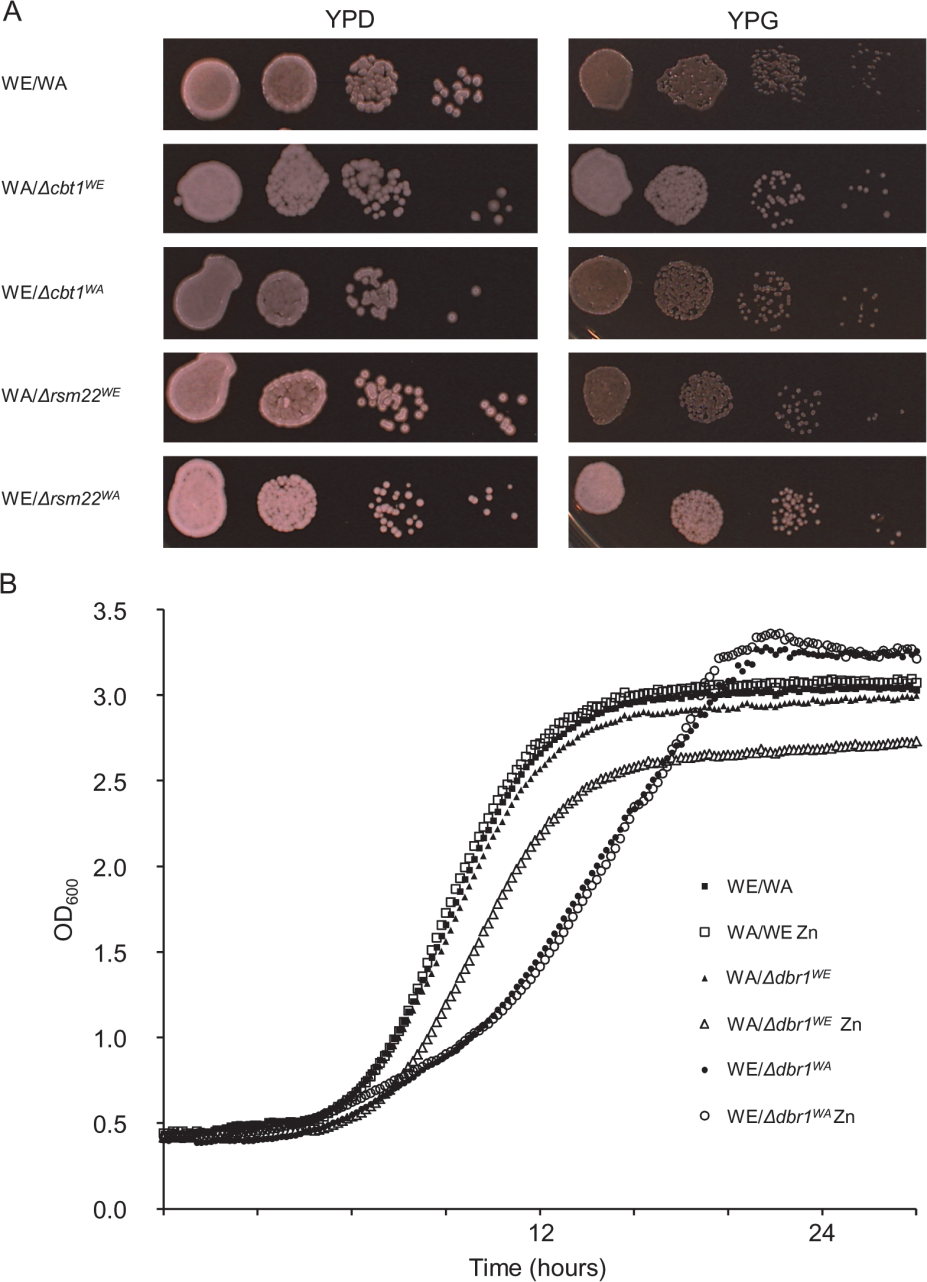
Phenotypic characterization of *CBT1*, *RSM22*, and *DBR1* alleles. A) *CBT1* and *RSM22* alleles did not show respiratory deficiency. Serial dilutions of heterozygous and hemizygous strain cells were plated on YPD medium and YPG medium containing 2% glycerol, which were grown at 28°C for one and two days, respectively. B) The hybrid carrying *DBR1* ^*WA*^ shows defective competitive fitness. Optical density at 600 nm (OD_600_) was monitored every 10 min as a growth measure at 28°C of the strains in SD medium and SD medium containing 3.5 mM ZnCl_2_.

From a gene pool identified after a large-scale functional analysis of respiratory-deficient yeast, the mutant Δ*cbt1 and Δrsm22* strains showed impaired respiratory performance [39]. The mitochondrial small ribosomal subunit protein Rsm22 participates in mitochondrial mRNA translation, and Cbt1 is involved in mt mRNA stabilization. Both of these proteins are essential for respiratory growth. To assess the putative effects of these alleles on respiration activity, serial dilutions of the wild-type, WA/Δ*cbt1* ^*WE*^, WE/Δ*cbt1* ^*WA*^, WA/Δ*rsm22* ^*WE*^, and WE/Δ*rsm22* ^*WA*^ strains were plated on YPD and YPG media and incubated at 28°C for 24 h and 48 h. No significant differences in growth were observed between the different hybrids on YPG medium with glycerol as the respiratory carbon source (Fig. 5A), suggesting that the *CBT1* and *RSM22* alleles do not significantly affect the respiratory activity of hybrid cells. Therefore, both the hybrid carrying *CBT1* ^*WE*^ and the hybrid carrying *RSM22* ^*WA*^ enhance dehydration tolerance with no apparent variation in cellular respiration.

### The *DBR1* ^*WA*^ allele provides competitive disadvantages to yeast cells

The RNA lariat debranching enzyme Dbr1p is involved in intron turnover, which is required for efficient Ty1 transposition [40]. The phenotypes already described for the Δ*dbr1* strain include decreasing competitive fitness and lower resistance to zinc deficiency. [41, 42]. We aimed to ascertain the growth performance of the Δ*dbr1* hemizygous strains in minimal medium and minimal medium supplemented with 1 μM, 3.5 mM, or 7 mM zinc dichloride (Fig. 5B shows the growth with 3.5 mM ZnCl_2_). Hybrids carrying *DBR1* ^*WA*^ and *DBR1* ^*WE*^ exhibited doubling times (DT) that were 5.8 min and 67.7 min higher, respectively, than the WE/WA strain. Both the hybrid carrying *DBR1* ^*WE*^ and the reference strain showed similar DT in media with or without Zn, but the hybrid carrying *DBR1* ^*WA*^ exhibited a 24.8 min higher DT in the presence of Zn than when grown in minimal medium alone.

## Discussion

Most of the genetic determinants of dehydration tolerance in yeast are still unknown. In this paper, two dehydration-tolerant QTLs were identified using a segregating population. By analysing strains with deleted genes in each QTL and by reciprocal hemizygosity assays, six genes have been confirmed to affect the capacity of yeast cells to survive dehydration and rehydration, namely the *BUD27*, *FAB1*, and *ATG18* genes mapped to QTLs on chromosome VI and the *CBT1*, *RSM22*, and *DBR1* genes in QTLs on chromosome XI. Furthermore, their phenotypic effects have been estimated. The genes *ATG18*, *RSM22*, and *DBR1* were not found to be necessary for desiccation tolerance in yeast cells [3, 10]. The fact that the genes mapped in our results do not fully coincide with previous genetic studies carried out with the *S*. *cerevisiae* deletion libraries of mutants sensitive to dehydration stress may indicate that different cellular mechanisms for overcoming stress imposition were caused by dissimilar selective forces exerted during the evolution of the yeast strains, or because the mutations present in the laboratory strains used for these studies are the effectors of these particular phenotypes [43–46]. Therefore, small discrepancies among the genes associated with cell dehydration tolerance from different studies support the idea that different allelic combinations exert different effects.

The nitrogen-deficient sporulation medium contains acetate as a carbon source to promote high levels of respiration, which induce sporulation in diploid yeast strains. In *S*. *cerevisiae*, the Δ*atg18* mutant is defective in sporulation but does not exhibit impaired vacuolar acidification [33]. The sequences of the *ATG18* ^*WA*^ and *ATG18* ^*WE*^ alleles revealed seven non-identical nucleotides. However, only one point mutation at nucleotide 584, from G to A, causes a single amino acid change of a serine to an asparagine residue (S195N; Table 2). Multiple sequence alignment of the WE and WA ATG18 alleles with 25 sequences of the *ATG18* gene from different *S*. *cerevisiae* strains annotated in the *Saccharomyces* Genome Database (SGD), as well as the Atg18 sequence characterized in this study, showed that the S residue is present in 16 genes, the N in eight genes, and the R in only one. This residue is located in the N-terminal region before the two WD40 domains and within a patch of highly conserved residues in Atg18 from *Pichia pastoris*, *Schizosaccharomyces pombe*, and *H*. *sapiens* [47]. The immediate response of yeast cells to osmotic challenge involves the release of calcium from the vacuole and the formation of fragmented vacuoles [48]. Our results suggest that the *FAB1* ^*WE*^ allele principally affects vacuolar morphology, which might allow the hybrid carrying *FAB1* ^*WE*^ to adapt quickly to ionic stress. However, 1 M sorbitol osmotic stress at 37°C is lethal to these cells when the WE/WA strain and the hybrid carrying *FAB1* ^*WA*^ are adapted. The *FAB1* ^*WA*^ and *FAB1* ^*WE*^ allele sequences revealed 15 non-identical nucleotides, producing differences in 12 residues (Table 2); however, only the N1273D and Y1300H mutations are located in a region of conserved residues within the Zn-finger domain [49]. Furthermore, none of these residues have a high identity ratio among the Fab1 sequences from the 28 *S*. *cerevisiae* strains (SGD). Fab1 governs vacuole homeostasis by generating PtdIns(3,5)*P*_*2*_ on the vacuolar membrane. Atg18 colocalizes with Fab1, and its deletion causes an abnormal elevation in the levels of PtdIns(3,5)*P*_*2*_, which suggests that Atg18 is also a negative regulator of the Fab1 kinase pathway [31]. The hybrid carrying *FAB1* ^*WA*^ and the hybrid carrying *ATG18* ^*WE*^ exhibit an osmotic pressure-dependent growth phenotype (Fig. 4C), indicating that the genes are essential for growth only at high temperatures in the presence of osmotic ionic stress. At the permissive temperature, the hybrids carrying *FAB1* ^*WA*^ and the hybrid carrying *ATG18* ^*WE*^ exhibited extremely defective growth. These phenotypes are comparable to the ones exhibited by some of the temperature-sensitive isolated vacuolar protein sorting (*vps*) mutants, which require one or more vacuolar functions at the permissive temperature that cannot be provided at 37°C by other vacuolar components in these mutant cells [50].

The *DBR1* gene is conserved in humans (*hDBR1*) and maintains the same function in both human and yeast cells [51]. Among other phenotypes of the Δ*dbr1* strain, decreases in competitive fitness and Zn deficiency stress resistance have been previously described [41–42]. The growth fitness of a strain with the *DBR1* ^*WE*^ allele is affected and this strain is less sensitive to Zn stress than the *DBR1* ^*WA*^ allele, for which the opposite effect on growth is observed. The *DBR1* ^*WA*^ allele had K^286^ and N^325^ residues in the putative HMM domain, replacing E^286^ and D^325^, respectively (Table 2), which are 100% conserved in other Dbr1 peptides deduced from the genomic sequences of 26 different *S*. *cerevisiae* strains (SGD). The deduced sequence of Cbt1^WA^ showed two residue differences with Cbt1^WE^, S29G, and T109A. In addition, three mutations were observed between the deduced peptide sequences of the *RSM22* ^*WA*^ and *RSM22* ^*WE*^ genes: E228K, D474S, and S619N (Table 2). These mutations do not affect the respiratory capacity of the different strains, thus enabling the separation of dehydration stress tolerance from respiration capacity. However, the above-mentioned variations in the sporulation efficiency of the *ATG18* hemizygous strains are not due to a pleotropic effect of the *RSM22* or *CBT1* alleles that affects cellular respiration.

The genetic approach used in this study, with a population of 96 segregants, allowed the detection of yeast dehydration resistance QTLs. The *RSM22* and *ATG18* genes enclosed within these QTLs that provide dehydration tolerance to the cell were not referenced in previous studies. Additionally, a detrimental effect on dehydration stress tolerance was shown to be provided by *DBR1* gene products. Our results further the understanding that dehydration stress tolerance is not a phenotype that results from the individual addition of independent genes. Furthermore, the monogenic approach is not suitable for summarizing all of the epistatic effects driven by a group of alleles. Currently, the successful long-term storage of living cells is of critical importance, but the frequently contradictory results associated with complex eukaryotic cells make the application of a simpler model system desirable. There are a number of advantages, including ease of growth and modification and well-characterized cell physiology, genetics and biochemistry, which make yeast cells the model of choice for anhydrobiotic engineering.

## Supporting Information

S1 Fig Diagrams of strain generation. A) Production of F1 population [52]. B) Haploid strains were disrupted for the identified genes (e.g., *ATG18*) using *URA3* and used to develop heterozygous diploid strains by reciprocal hemizygous crossover.
(TIF)

S2 Fig Quantitative real-time PCR analysis of gene expression before stress. Data represent mean relative expression ± SD (y axis, Log2 values) of each individual gene (show at the bottom) before dehydration of different strains. Genes *ALG9* and *TAF10* were simultaneously used as constitutive reference genes as determined by the geNorm algorithm [53]. Relative expression was calculated using REST-MCS v2 software [54].
(TIF)

S1 Table Primers used in this study. (DOCX)
