# Supplementary figures and images for: ATG18 and FAB1 Are Involved in Dehydration Stress Tolerance in Saccharomyces cerevisiae
Source: PLoS One. 2015 Mar 24;10(3):e0119606. doi: 10.1371/journal.pone.0119606 (PMC4372426; doi:10.1371/journal.pone.0119606)

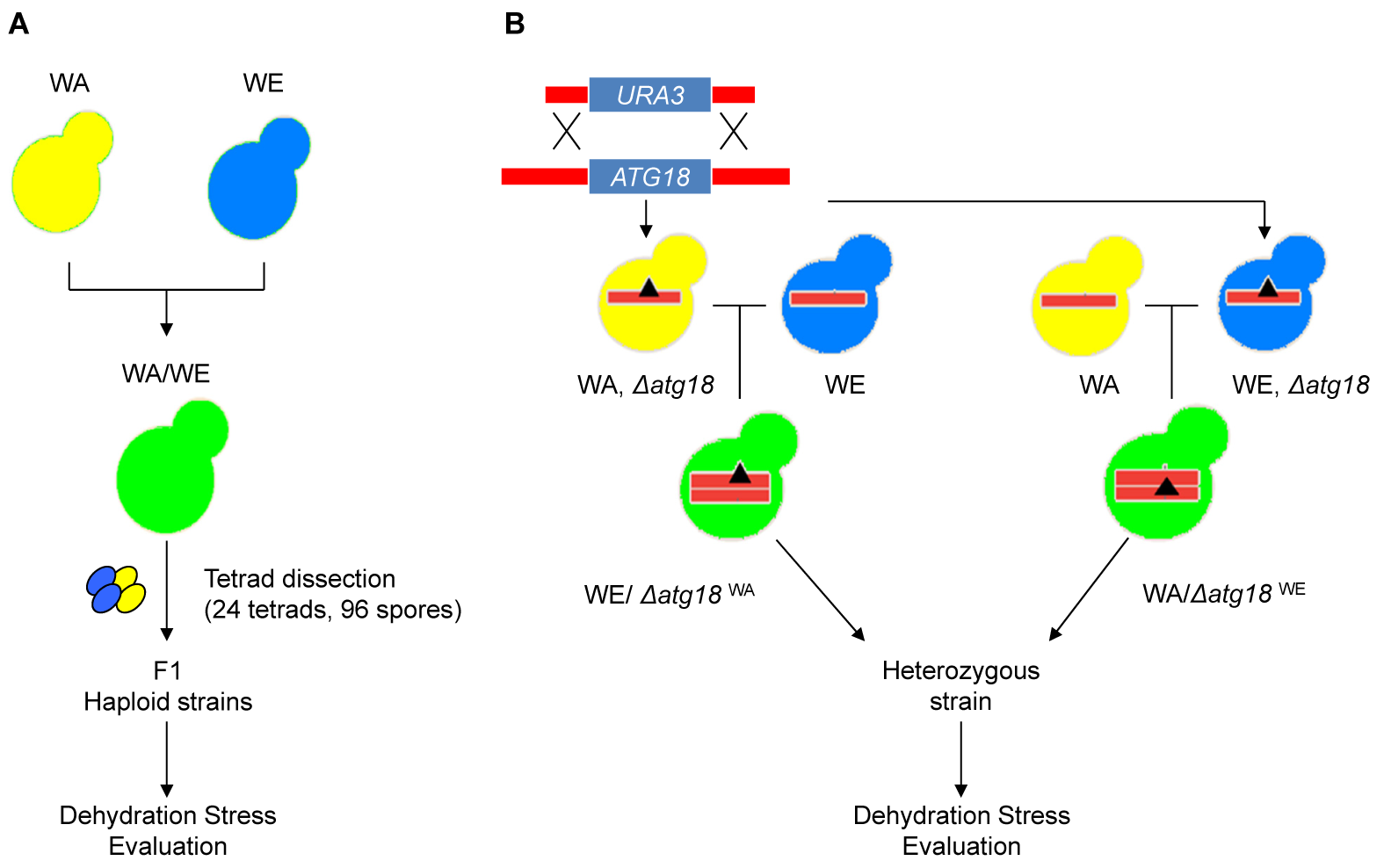

Supplement: S1 Fig — A) Production of F1 population [52]. B) Haploid strains were disrupted for the identified genes (e.g., ATG18) using URA3 and used to develop heterozygous diploid strains by reciprocal hemizygous crossover. (TIF) [file pone.0119606.s001.tif]

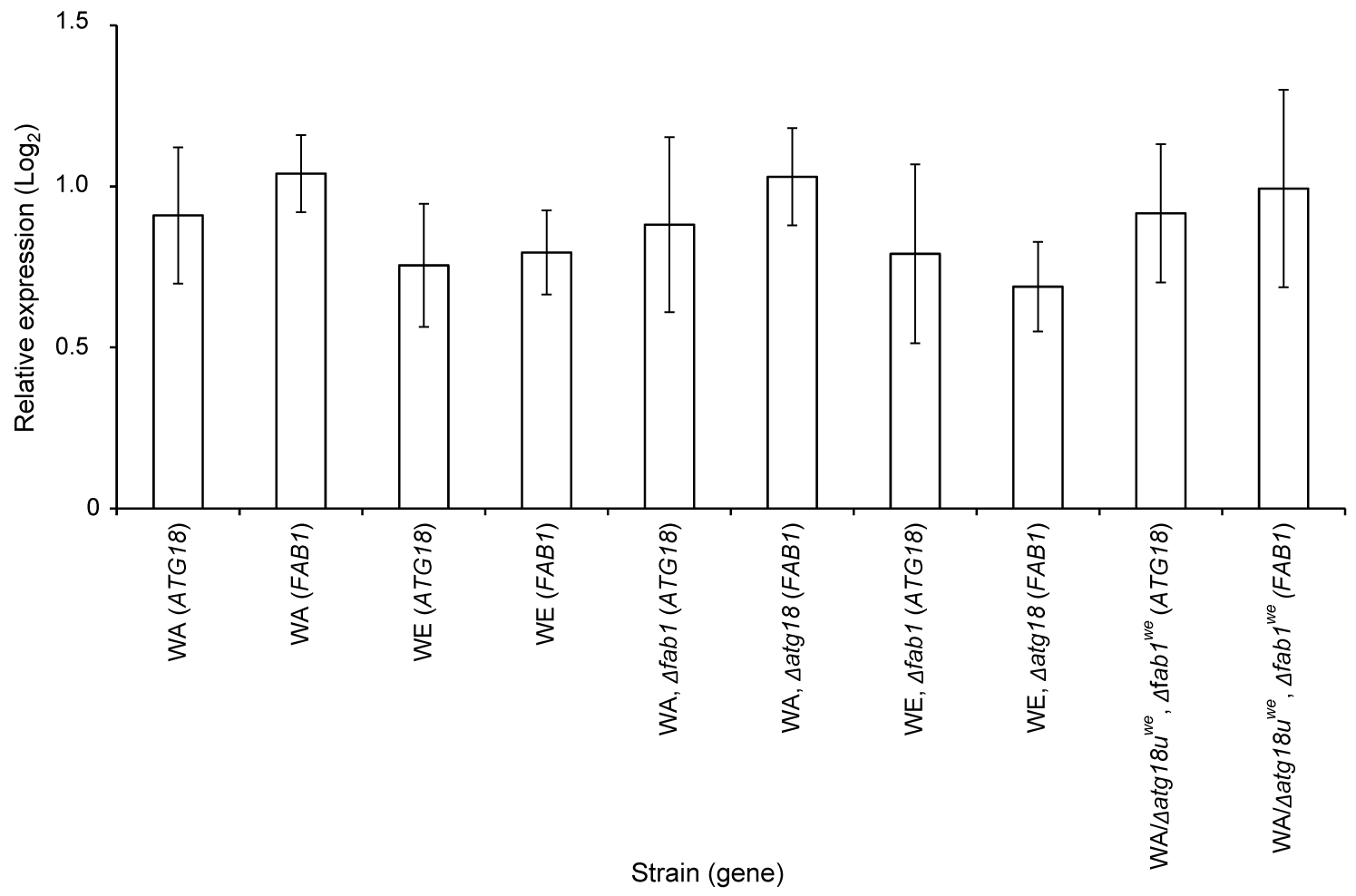

Supplement: S2 Fig — Data represent mean relative expression ± SD (y axis, Log2 values) of each individual gene (show at the bottom) before dehydration of different strains. Genes ALG9 and TAF10 were simultaneously used as constitutive reference genes as determined by the geNorm algorithm [53]. Relative expression was calculated using REST-MCS v2 software [54]. (TIF) [file pone.0119606.s002.tif]
