# Supplementary material for: ATG18 and FAB1 Are Involved in Dehydration Stress Tolerance in Saccharomyces cerevisiae
Source: PLoS One. 2015 Mar 24;10(3):e0119606. doi: 10.1371/journal.pone.0119606 (PMC4372426; doi:10.1371/journal.pone.0119606)
Supplement: S1 Table — (DOCX) [file pone.0119606.s003.docx]

Table S1 Primers used in this study

| Primer | Nucleotide sequence (5'-3') |
| --- | --- |
| HXT10fw | ACGAAATCCCATACGCAAAT^1^ |
| HXT10rv | AGCCGATGAGATGAGGATTG^1^ |
| ARS605fw1 | CCATGTAGCTTATCGCAGCA^1^ |
| ARS605rv1 | CCAAGCAGTTCTCTAGCTCCA^1^ |
| ARS605fw2 | TGGCACTTCGTATATGCAACA^1^ |
| ARS605rv2 | AGGAACCAAAAATCGCCAAC^1^ |
| YFR016Cfw2 | TGACCTTTCAATTTAGAAGATTTCC^1^ |
| YFR016Crv2 | CTGGTGTGCTTGATTACTCTGG^1^ |
| ARS1103fw | TGGGCTATTTCATCCCATTG^1^ |
| ARS1103rv | GAGAAGGAGGCAGCAGGAG^1^ |
| YKT6fw | CGCCACCCAATAAGAAAAA^1^ |
| YKT6rv | CTTGTTTGGTGTCGGCATAA^1^ |
| TP05fw | TCGTCAGCTAAAGCAGGTAACA^1^ |
| TP05rv | ACAAAGTCTGTGTTTATTGGATCA^1^ |
| PIR1fw | TGAGAATTGTAGCATTACGATCTTCT^1^ |
| PIR1rv | GCCTTTTTATGTCCTGCCAAT^1^ |
| PIR3fw1 | AGGTATGTGCGCAGCTCTTT^1^ |
| PIR3rv1 | CCGGTGACCTTACCGATCTA^1^ |
| PIR3fw2 | GAGCAACCATTTCCGAATGT^1^ |
| PIR3rv2 | ACGGCGAAATATGCCAAA^1^ |
| RIM15fw | ATTATTCTCAGATTTGCTTTCAAGCAAAGTTTTTATTCAGTTATTTTTTTTAATTATCTTTATCTTAAAATTTATAGCTTTTCAATTCAATTCATCAT^1^ |
| RIM15rv | AGCATTTTCCCTTTTTTTTTTTCCCTTTCCTTTCTCTTGCCTCATTTGATAGAATAGATAAGCCCAGTAGAGGAAGACAGAAGCTTTTTCTTTCCAATT^1^ |
| RIM15A1fw | ATTCTGCTTTTAATATTTCCAGATTT^1^ |
| BST1fw | CAAGCTTTTTTCTTGCCATGATCTAGAACTCTCAGGCAATATATACAGTTAATCTTTTTTTACTGGGTTGTAGTTCAGCTTTTCAATTCAATTCATCAT^1^ |
| BST1rv | CACACTCGAAATACTCCCTCTACTTTAAAGCATTGGCCTATATCTTAGGCTTACCATCATACAAAAATCTTCATTTCGTTAAGCTTTTTCTTTCCAATT^1^ |
| BST1A1fw | TGATCAAAATTTACGGCTTTGA^1^ |
| BUD27fw | GAATTTTATAGTAAACAGGTATCCTCAGACTGTAATAGCCAAGCTTTTCAATTCAATTCATCAT^1^ |
| BUD27rv | GTTAATATAGATTCTGATTTACTTTCTGTCTCCATATGGGTAGCTTTTTCTTTCCAATT^1^ |
| BUD27A1fw | AATTTTGTGGTCGGATCGTG^1^ |
| BLM10fw | TGTATTTGCATACATAAACTTTATCATTGTTCGTTAGCTAGCTTTGCACATTAATTTTTCGATTTGTTACCGCCAAAGCTTTTCAATTCAATTCATCAT^1^ |
| BLM10rv | AATCAGCAGATAGCTCCAGCTATTTGTTTAGATGTACATATATGTCTAGATATGTGCTTAATATCCTATACTAATATGAATAGCTTTTTCTTTCCAATT^1^ |
| BLM10A1fw | GGCCGAGGTATCCCTTAGAA^1^ |
| YFH7fw | AACCTTGTTAGGTTAATTTCACTAGTACTATACATATTTTATCCTGTATCATACCAGAGGATCATTCTAGCCACAAAGCTTTTCAATTCAATTCATCAT^1^ |
| YFH7rv | TGCCGTTTTTGCTTGCGCGCCTTAATTATCTGTATTCAGTTCGATTTTACAAAAATATATACAAGGTTCCGCCTAACCTTCAGCTTTTTCTTTCCAATT^1^ |
| YFH7A1fw | TGGGCTTATCAGACTTGTCCA^1^ |
| FAB1fw | ATAAAGGGCCAAACAAAAAACTATTTCGAATAGCAAGGTAGCTTCCATCCTGTACATGCAAGACCGTCACACAGCAAGCTTTTCAATTCAATTCATCAT^1^ |
| FAB1rv | TACTGAAAGTTAAAGAACACTAATGTGCGTGATAGTGTATAAAAAAAAGTTACAGAATATAACTTGTACACGTTTATGTATAGCTTTTTCTTTCCAATT^1^ |
| FAB1A1fw | TAACTCTCCCTCTCCCCCTCT^1^ |
| ATG18fw | CACGACCCTCCCTTATTAATCAGTTAGTAATAGTGTTCCAGTTAACTCTGTATCCTTTTCTTCTTCGGCCTGACAAAGCTTTTCAATTCAATTCATCAT^1^ |
| ATG18rv | AGATTATACGCAGGAGTTTATATAAACTATATTGTGTATGCGTTGTGACGTACGGAAGGCAGCGCGAGACACTTCCGTGATAGCTTTTTCTTTCCAATT^1^ |
| ATG18A1fw | CATTCGGAAGTGCGACAATA^1^ |
| RPL2Afw | ACAATCACATGGTTGTTAAATCACGGTGCTGACATACCCATAGCTTTTCAATTCAATTCATCAT^1^ |
| RPL2Arv | GAACTGGTTTGTTACGTGGTTCTCAAAAGACCCAAGATTAGAGCTTTTTCTTTCCAATT^1^ |
| RPL2AA1fw | AACTTGGCAGCACCTTGTCT^1^ |
| CBT1fw | GATTGATCAGAAGTTTACTGCGCTTTTGGGTAAAGAAGCATTAAACAAAGGAGAGAGAAATATTGCAAGGAAAAAAAGCTTTTCAATTCAATTCATCAT^1^ |
| CBT1rv | AGCCAGTGCTATAGTCACCAAATAATACGCATTATATATGGATATGTACAGTTCGCAGATCTTTATGGCATATTTATCGTTAGCTTTTTCTTTCCAATT^1^ |
| CBT1A1fw | GCCATTTGCCTATAGCTTGG^1^ |
| MRP49fw | AGTTTTGAATTTACATATTTCCATGAAGGGCAATGTTTTTTGATATATACATGAACAAACTTATCGAGAGAAAGCTAGCTTTTCAATTCAATTCATCAT^1^ |
| MRP49rv | CAGGATATCTGTAAGAATCGGCCATAAAACTCATTAATAGAAGAACAGTATAACATAAGTGAGCCTGCTACAATAAGAAGAAGCTTTTTCTTTCCAATT^1^ |
| MRP49A1fw | TCTCCTCCTGCATTACCATTG^1^ |
| RSM22fw | ATATTCACGTATGTAGAATATTAAAGTATTGAATATATTAATATTATTACTTTATTTCCAGTTACTTACAATTTCCAGCTTTTCAATTCAATTCATCAT^1^ |
| RSM22A1fw | GTTACCTGCGAATCCTGCTC^1^ |
| DBR1fw | GTATGACTAAAAATTCTCTCAAGAAGGCTTGGCTTTAAGCTCTAATTCCGTCTGCATTCGTAATAGAAATATCTCTAGCTTTTCAATTCAATTCATCAT^1^ |
| DBR1rv | AAATGAGCAGGAGAAAGTCATATGGCGAACGTAAATATGTAACTAAAAATTAAGATGGGCAGACATTTATCATTTTGCTTAAGCTTTTTCTTTCCAATT^1^ |
| DBR1A1fw | GTCCCCCACCATTTATGAAC^1^ |
| ATGufw | CACGACCCTCCCTTATTAATCAGTTAGTAATAGTGTTCCAGTTAACTCTGTATCCTTTTCTTCTTCGGCCTGACAAatcacggaagtgtctcgcgctgc |
| ATGurv | AGATTATACGCAGGAGTTTATATAAACTATATTGTGTATGCGTTGTGACGTACGGAAGGCAGCGCGAGACACTTCCGTGATttgtcaggccgaagaag |
| S8rv | CCTCTAGGTTCCTTTGTTACTTCT^2^ |
| MATaspe | ACTTCCACTTCAAGTAAGAGTTTG^3^ |
| MATαspe | GCACGGAATATGGGACTACTTCG^3^ |
| MATfla | AGTCACATCAAGATCGTTTATGG^3^ |
| ATG18fw | GAAACTTCCCGTTGAAACCA^1^ |
| ATG18rv | CCGGATACTCGGATGTGTCT^1^ |
| FAB1fw | TTGATCGCATTTTGCTTGAG^1^ |
| FAB1rv | TTGGGCATTCAAGTTCATCA^1^ |
| ALG9fw | GCCGTCTACGAGCAATTTTC^1^ |
| ALG9rv | TCTGGCAGCAGGAAAGAACT^1^ |
| TAF10fw | CCAGGATCAGGTCTTCCGTA^1^ |
| TAF10rv | AGCTCTCGCCTGACTGTTGT^1^ |

Primer´s references: ^1^this work, ^2^52, ^3^21
